# *PLP1* gene mutations cause spastic paraplegia type 2 in three families

**DOI:** 10.1002/acn3.51722

**Published:** 2023-01-09

**Authors:** Li Yao, Zeyu Zhu, Chao Zhang, Wotu Tian, Li Cao

**Affiliations:** ^1^ Department of Neurology Shanghai Sixth People's Hospital Affiliated to Shanghai Jiao Tong University School of Medicine Shanghai 200233 China; ^2^ Suzhou Hospital of Anhui Medical University, Suzhou Municipal Hospital of Anhui Province Suzhou 234000 China

## Abstract

**Objective:**

Spastic paraplegia type 2 (SPG2) is an X‐linked recessive (XLR) form of hereditary spastic paraplegia (HSP) caused by mutations in proteolipid protein 1 (*PLP1*) gene. We described the clinical and genetic features of three unrelated families with *PLP1* mutations and reviewed *PLP1*‐related cases worldwide to summarize the genotype–phenotype correlations.

**Methods:**

The three probands were 23, 26, and 27 years old, respectively, with progressively aggravated walking difficulty as well as lower limb spasticity. Detailed physical examination showed elevated muscle tone, hyperreflexia, and Babinski signs in lower limbs. Brain MRI examinations were investigated for all cases. *PLP1* mutations were identified by whole exome sequencing, followed by Sanger sequencing, family co‐segregation, and phenotypic reevaluation.

**Results:**

A total of eight patients with SPG2 were identified in these three families. The probands additionally had cognitive impairment, urinary or fecal incontinence, ataxia, and white matter lesions (WML) in periventricular regions, with or without kinetic tremor. Three hemizygous mutations in *PLP1* were identified, including c.453+159G>A, c.834A>T (p.*278C), and c.434G>A (p.W145*), of which c.834A>T was first associated with HSP.

**Interpretation:**

We identified three families with complicated SPG2 due to three *PLP1* mutations. Our study supports the clinically inter‐and intra‐family heterogeneity of SPG2. The periventricular region WML and cognitive impairment are the most common characteristics. The kinetic tremor in upper limbs was observed in 2/3 families, suggesting the spectrum of PLP1‐related disorders is still expanding.

## Introduction

Hereditary spastic paraplegia (HSP) is a highly clinically and genetically heterogeneous group of neurodegenerative diseases characterized by progressive spasticity of the lower limbs.^1^, ^2^ They are characterized by length‐dependent corticospinal tract and dorsal column degeneration with a prevalence ranging from 0.1 to 9.6/10^5^ around the world.^3^ Currently, up to 101 genetic loci and 86 subtypes have been described in HSP, which can be categorized into pure or complicated forms on the basis of clinical features.^4^, ^5^

In 1957, Blumel et al.^6^ first reported a family of X‐linked recessive (XLR) spastic paraplegia. Subsequently, Keppen et al.^7^ demonstrated the location of the locus for this disorder, designated Spastic paraplegia type 2 (SPG2, OMIM # 312920), in the middle of the long arm of the X chromosome. Saugier‐Veber et al.^8^ found that proteolipid protein 1 (*PLP1*, NM_000533) is a possible candidate gene for SPG2 by narrowing the genetic interval in the X‐linked SPG family reported by Bonneau et al.^9^ SPG2 is a rare subtype of XLR‐HSP due to mutations in *PLP1* gene. Therefore, males who carry a *PLP1* pathogenic variant are mostly affected. However, neurological symptoms are occasionally observed in some female carriers.^10^, ^11^ Clinical phenotypes of SPG2 compromise pure and complicated forms usually occurring in the first decade of life.^11^ The complicated form is characterized by additional neurological dysfunctions, such as dysarthria, ataxia, cognitive impairment, and nystagmus.^12^, ^13^

Here, we described the clinical and genetic features of three families with SPG2, and further summarized the genotype–phenotype correlations.

## Material and Methods

### Participants

We identified three probands fulfilling the diagnosis of HSP according to progressive spasticity of lower limbs and walking difficulty.^4^ All probands and their family members were clinically examined.

### Ethical approval

Written informed consent was obtained from the patients. The ethics committee of Shanghai Sixth People's Hospital Affiliated to Shanghai Jiao Tong University School of Medicine approved the study.

### Mutation analysis

Genomic DNA was extracted from peripheral blood lymphocytes with a standard phenol/chloroform extraction protocol. Healthy individuals (*n* = 300) of matched geographic ancestry were included as normal controls. Exome sequencing was performed for the patients, using Agilent SureSelect v6 reagents for capturing exons and Illumina HiSeq X Ten platform. Alignment to human genome assembly hg19 (GRCh37) was carried out followed by recalibration and variant calling. Population allele frequencies compiled from public databases of normal human variation [1000 Genomes (1000 g; http://browser.1000genomes.org), the Exome Aggregation Consortium (ExAC; http://exac.broadinstitute.org), dbSNP (https://www.ncbi.nlm.nih.gov/projects/SNP/), NHLBI Exome Sequencing Project (ESP) Exome Variant Server (http://evs.gs.washington.edu/EVS), and the Genome Aggregation Database (gnomAD; http://gnomad‐sg.org/)] were used to initially filter the data to exclude variants at >1‰ frequency in the population. The variants were further interpreted and classified according to the American College of Medical Genetics and Genomics (ACMG) Standards and Guidelines.^14^ In this segment, two neurogeneticists analyzed the inheritance pattern, allele frequency (from: 1000 g, ExAC, dbSNP, gnomAD, NHLBI Exome Sequencing Project (ESP) Exome Variant Server, and 300 healthy controls), amino acid conservation, and nucleotide pathogenicity prediction [Mutationtaster (http://www.mutationtaster.org), PolyPhen‐2 (http://genetics.bwh.harvard.edu/pph2/), and Scale‐invariant feature transform (SIFT; http://sift. jcvi.org)]. The variants were further interpreted and classified according to the ACMG guidelines.^14^ Putative pathogenic variants were further confirmed by Sanger sequencing both forward and reverse strands.

## Results

### Clinical findings

**Figure 1:**
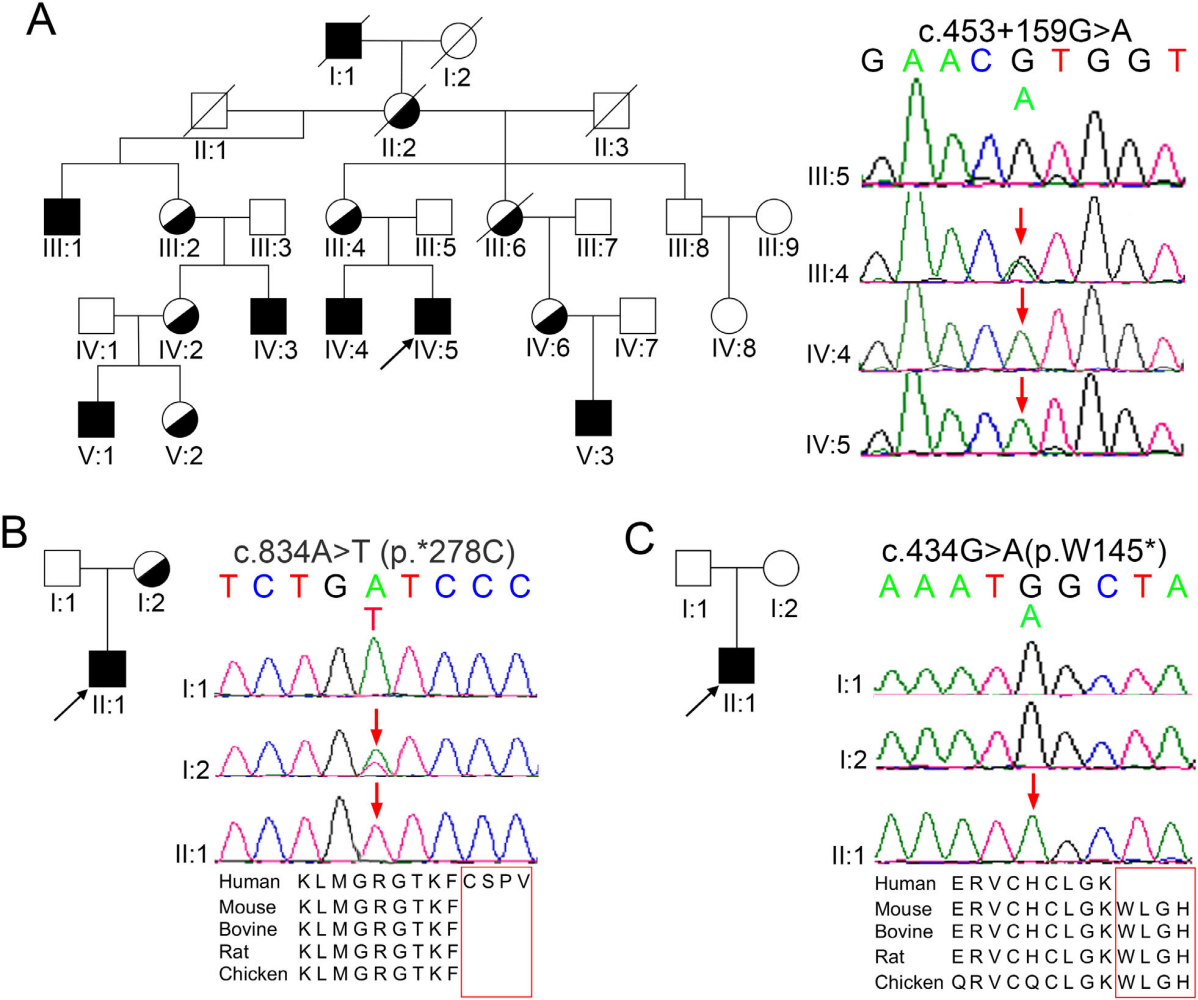
Pedigree of three families with SPG2 and conservation analysis of the *PLP1* mutations among different species. The pedigree is shown with squares representing males, circles representing females; black‐filled symbol representing affected, the white symbol representing unaffected, and the half white and half black symbol representing heterozygous carriers, respectively. (A) Pedigree of Family 1. Sequence chromatogram of PLP1 gene displays one hemizygous intronic mutation c.453+159G>A in the proband (IV:5), which was identified in his mother (III:4) and affected brother (IV:4) but negative in his father (III:5). (B) Pedigree of Family 2. Sequence chromatogram of PLP1 gene displays one hemizygous elongation mutation c.834A>T (p.*278C) in the proband (II:1), which was identified in his mother (I:2) but negative in his father (I:1). (C) Pedigree of Family 3. Sequence chromatogram of PLP1 gene displays one hemizygous de novo mutation of c.434G>A, p.W145* in the proband (II:1), which was negative in both parents (I:1 and I:2). (B,C) The mutations located in the highly conserved region of proteins are shown in the bottom half. Red square frame: mutant amino acid.

**Figure 2:**
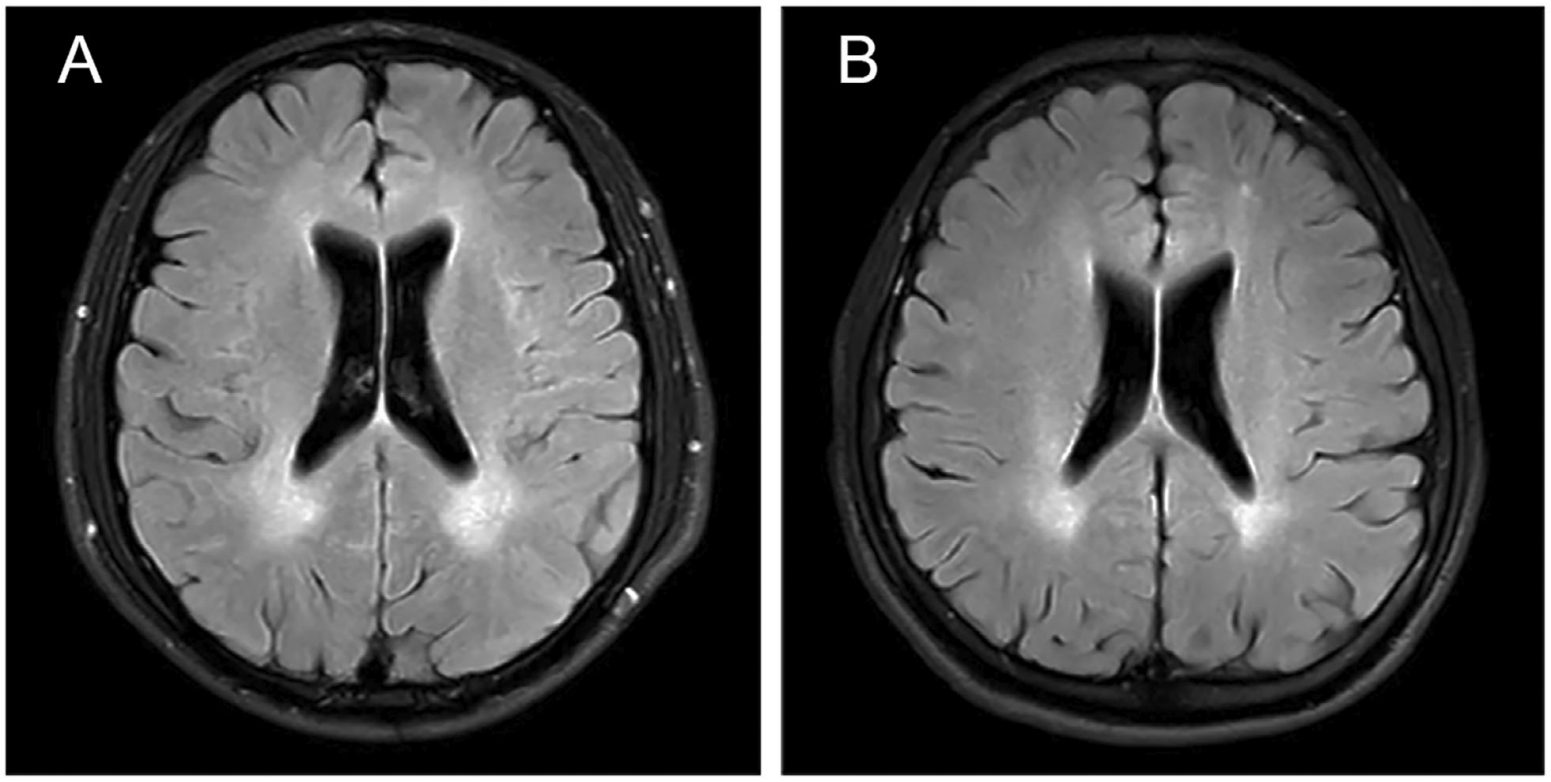
Brain MRI of patients with SPG2. (A) (patient T1866) and (B) (patient T6956) showed hypersignal intensity of white matter in periventricular regions on Flair‐weighted sequences.

Family 1 was comprised of five generations including six male patients presenting with progressive muscle weakness and spasticity in lower limbs. The proband T1866 (IV:5 in Fig. 1A) was a 23‐year‐old male, with progressive gait disturbance for 5 years. During school days, poor performances of physical education examinations were recorded. His initial symptoms appeared at the age of 18 when he had difficulty in running and climbing stairs. Three years later, abnormal walking posture and pes valgus were noticed. His symptoms progressively aggravated and mild cognitive dysfunction was noted at the age of 23. Physical examination showed hyperreflexia, weakness (4/5 on a medical research council scale graded 0–5), bilateral ankle clonus, and Babinski signs in lower limbs. At the age of 23, he was able to walk alone slowly with a scissors gait and occasionally experienced urinary incontinence. He scored 10 points in Spastic Paraplegia Rating Scale (SPRS). Magnetic resonance imaging (MRI) of the brain showed white matter lesions (WML) in the periventricular regions (Fig. 2A). Nerve conduction studies showed impairment of deep sensory pathways in both lower extremities. The similar symptoms and physical examination results of his 29‐year‐old brother T2137 (IV:4 in Fig. 1A) were recorded. However, he manifested with a more complicated and much severer phenotype, including platypodia, sensory disturbance of distal extremities, kinetic tremor in upper limbs, delayed motor milestones, mental retardation after birth, and schizophrenia at the age of 17. Right now, he is still able to walk alone slowly with a scissors gait without assistance. He scored 17 points in SPRS.

In Family 2, the proband T6956 (II:1 in Fig. 1B) was a 26‐year‐old male with progressive unsteady gait and leg stiffness for 5 years. He had severe ichthyosis for 19 years and hyperuricemia for 4 years. The physical examination showed hyperreflexia, weakness (4/5), bilateral ankle clonus, and Babinski signs in the lower limbs. Recently, he complained about a moderate decline in recent memory. At the most recent outpatient visit, he was able to walk independently on flat ground with a scissors gait. He scored 12 points in SPRS. The results of MRI showed WML in the periventricular regions (Fig. 2B) and multiple Schmorl's nodes in lumbar vertebrae.

In Family 3, the proband T0650 (II:1 in Fig. 1C) was a 27‐year‐old male with gait disturbance. Leg weakness, drag‐to and toe‐walking gait were first noted between the age of 2 and 3 years old. Later on, he had kinetic tremor in upper extremities, especially when taking chopsticks and fastening buttons. He had fecal and urinary incontinence since early childhood. Neurological examination showed dysarthria, right horizontal nystagmus, and involuntary movements of lip with mild cognitive impairment. He had reduced strength (4/5), and increased muscle tone in his lower limbs, without muscle atrophy. Tendon reflexes were brisk in lower limbs with bilateral ankle clonus and bilateral Babinski signs. Abnormal results were disclosed bilaterally during the finger‐to‐nose test, heel–knee‐tibia test, and Romberg test. The patient was able to walk slowly with the help of walker with a scissor gait. Brain MRI showed symmetrical diffuse hyperintensity in bilateral paraventricular central semiovale, posterior limb of internal capsule, corpus callosum, bilateral cerebellopontine brachium conjunctivum, and medulla oblongata on T2‐weighted sequences.

The detailed clinical features of the four patients are all summarized in Table 1.

**Table 1:** Clinical features and *PLP1* gene mutations of patients with SPG2.

Family	Patient	Gender	AAO (years)	Duration (years)	Inheritance	Phenotype	UL	LL			Babinski signs	Ankle clonus	Additional features	SPRS	*PLP1* Variant	ACMG
								Hypertonia	Hyperreflexia	Weakness						
Family 1	T1866	M	18	5	XLR	C	−	+	+	+	+	+	CI; UI; valgus foot; WML.	10	c.453 + 159G>A	P
	T2137	M	0	29	XLR	C	Kinetic tremor	+	+	+	+	+	MD; platypodia; sensory disturbance; schizophrenia.	17	c.453 + 159G>A	P
Family 2	T6956	M	21	5	XLR	C	−	+	+	+	+	+	CI; proteinuria; hyperuricemia; ichthyosis; WML.	12	c.834A>T (p.*278C)	P
Family 3	T0650	M	3	24	S	C	Kinetic tremor	+	+	+	+	+	Dysarthria; nystagmus; CI; UI; FI; ataxia; WML.	20	c.434G>A (p.W145*)	P

AAO, age at onset; XLR, X‐linked recessive; S, sporadic; +, positive; −, negative; C, complicated; UL, upper limbs; LL, lower limbs; SPRS, Spastic Paraplegia Rating Scale; PLP1, proteolipid protein 1 (NM_000533); ACMG, American College of Medical Genetics and Genomics; M, male; CI, cognitive impairment; UI, urinary incontinence; FI, fecal incontinence; WML, white matter lesions; P, pathogenic; MD, mental retardation.

### Genetic findings

A hemizygote intronic variation c.453+159G>A in *PLP1* gene was identified in the proband (IV:5), his elder brother (IV:4) and their mother (III:4) which was negative in the unaffected father (III:5) of Family 1 (Fig. 1A). An elongation mutation c.834A>T (p.*278C) was disclosed in the proband (II:1) and his mother (I:2) but was negative in the unaffected father (I:1) of Family 2 (Fig. 1B). In Family 3, a *de novo* nonsense variant c.434G>A (p.W145*) was detected in the proband (II:1) which was negative in both parents (I:1 and I:2, Fig. 1C). The amino acid sites affected are all highly conserved among different species. All of the three variants were not identified in 300 healthy controls, 1000 Genome Project (http://browser.1000genomes.org), NHLBI Exome Sequencing Project (ESP) Exome Variant Server or ExAC, and were predicted as “disease causing” by multiple silicon software. c.434G>A has been recorded in dbSNP (rs132630292). According to ACMG guidelines,^14^ all the variants in *PLP1* genes are classified as “pathogenic” (Table 1).

## Discussion

PLP1‐related disorders include a wide spectrum of XLR neurodegenerative dysfunctions. So far, a total of 392 *PLP1* mutations have been reported to be associated with SPG2, multiple sclerosis, hypomyelination of early myelinating structures (HEMS), Pelizaeus–Merzbacher disease (PMD), autism, neurodevelopmental disorders, and early‐onset neurological disease trait (EONDT),^15^, ^16^, ^17^, ^18^, ^19^, ^20^, ^21^, ^22^, ^23^, ^24^, ^25^, ^26^, ^27^ which differ in the onset, severity of symptoms and neuroimaging findings.^17^ Among these, PMD typically manifests as severe spasticity, ataxia, nystagmus, hypotonia, cognitive impairment, WML, and shortened lifespan, usually with onset in infancy or early childhood.^28^ However, SPG2 patients usually have normal life span.^11^ HEMS represents an intermediate phenotype between PMD and pure SPG2.^29^ Among these, sever PMDs are usually due to duplication mutations and gross insertions,^30^ while milder forms, such as SPG and milder PMD, could be related with mutations in less conserved regions.^31^

**Figure 3:**
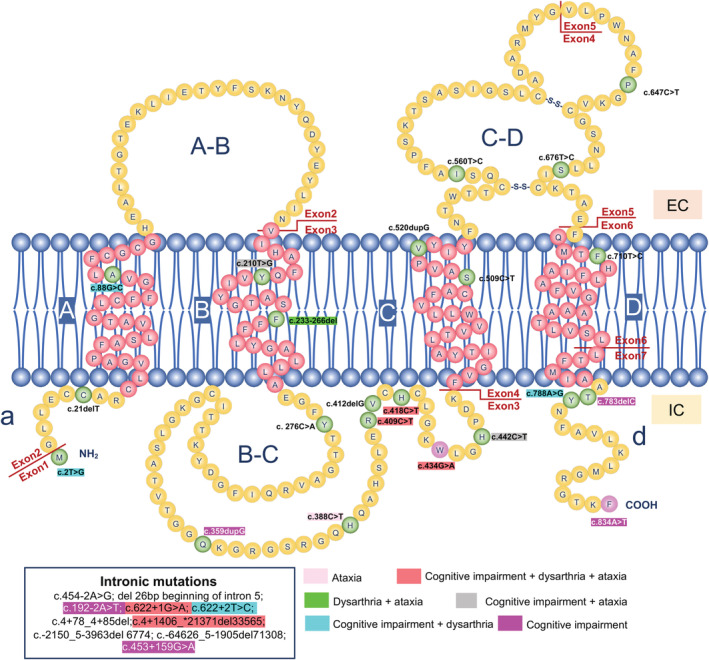
Schematic diagram of PLP1 structure and summary of genotype–phenotype correlations of SPG2. Mutation spectrum of SPG2. The schematic diagram of PLP1 structure with all mutations in exons associated with SPG2 were highlighted with different colors. Intronic mutations were listed in the left bottom. Genotype–phenotype correlations of SPG2 were highlighted with different colors. PLP1 is 30 kDa tetraspanin protein with ‐NH_2_ and ‐COOH termini in cytoplasm. Full length of PLP1 (NM_000533) contains four transmembrane domains [A (aa from 10–36); B (aa 64–88); C (aa 152–177); D (aa 234–260)] and five topological domains [a (aa 2–9); A,B (aa 37–63); B,C (aa 89–151); C,D (aa 178–233); d (aa 261–277)]. These mutations distributed in different domains, such as “a” (22.22%, 2/9aa), “A” (3.70%, 1/27), “A,B” (0%, 0/27), “B” (8.00%, 2/25), “B,C" (8/63, 12.70%), “C” (2/26, 7.69%), “C,D" (3/56, 5.36%), “D” (1/27, 3.70%), “d” (3/17, 17.65%). Yellow balls: amino acids in topological domains; red balls: amino acids in transmembrane domains; green balls: known mutations associated with SPG2; purple balls: mutations reported in this study; EC, extracellular; IC, intracellular; NH_2_, amino terminal; COOH, carboxyl terminal; aa, amino acids.

**Figure 4:**
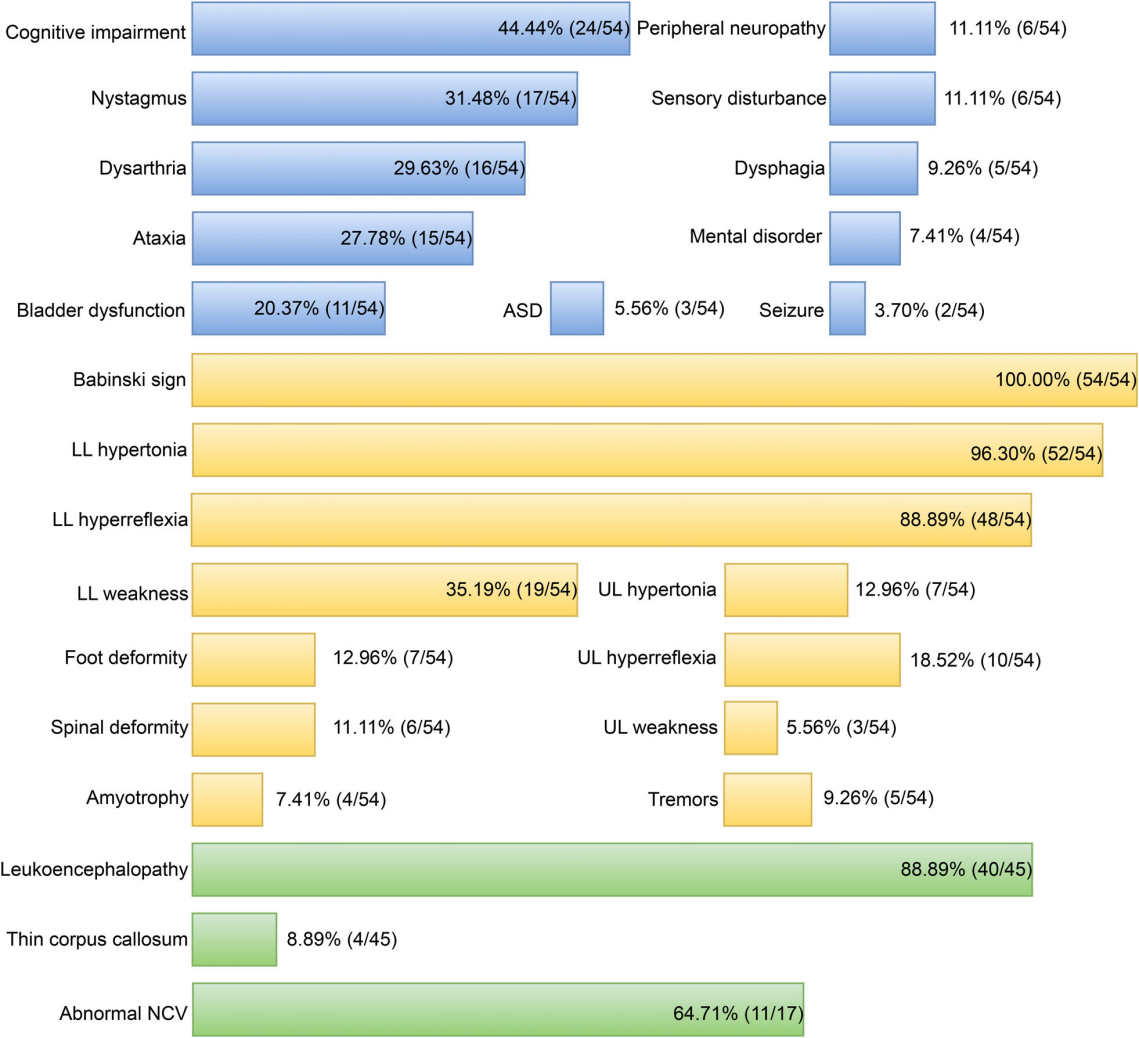
Clinical features of SPG2 patients with *PLP1* mutations. For each clinical feature, the proportion of patients is indicated. Blue: clinical symptoms; yellow: physical examinations; green: imaging and electrophysiological findings. UL, upper limbs; LL, lower limbs; ASD, anal sphincter dysfunction; NCV, nerve conduction velocity.

Up to now, 32 mutations have been documented to cause SPG2, including 12 missense mutations, 3 nonsense mutations, 7 frameshift mutations, 4 splicing mutations, and 6 deep intronic mutations, which distribute in different exons and introns, such as exon1 (1), exon2 (2), exon3 (10), exon4 (3), exon5 (2), exon6 (1), exon7 (3), and intronic regions (10) (Fig. 3). Intronic mutations included c.454‐2A>G, del 26 bp beginning of intron5, c.192‐2A>T, c.622+1G>A, c.622+2T>C, c.4+78_4+85del, c.4+1406_*2137del33565, c.‐2150_5‐3963del6774, c.‐64626_5‐1905del71308, and c.453+159G>A. The mutations in intron3 have been showed to alter PLP1/DM20 alternative splicing, resulting in the reduced PLP1/DM20 ratio.^27^, ^32^, ^33^ So far, a total of 36 SPG2 families have been reported worldwide, including 66 males and 16 females.^8^, ^10^, ^18^, ^31^, ^34^, ^35^, ^36^, ^37^, ^38^, ^39^, ^40^, ^41^, ^42^, ^43^, ^44^, ^45^, ^46^, ^47^, ^48^, ^49^, ^50^, ^51^, ^52^, ^53^, ^54^ SPG2 usually starts before age 10, while adult cases have also been reported.^45^, ^48^ All patients presented with gait abnormality (100%, 54/54). Moreover, complicated form is predominant (81.48%, 44/54), which is characterized by cognitive impairment (44.44%, 24/54), nystagmus (31.48%, 17/54), dysarthria (29.63%, 16/54), and ataxia (27.78%, 15/54). Physical examination showed Babinski sign (100%, 54/54), lower limbs hypertonia (96.30%, 52/54), and hyperreflexia (88.89%, 48/54) in lower limbs weakness (35.19%, 19/54). In MRI, thin corpus callosum (8.89%) and leukoencephalopathy (88.89%) are common neuroimaging findings, which mostly involve the periventricular regions, parieto‐occipital, internal capsule, corpus callosum, subcortical, medulla, thalamus, and brainstem.^10^, ^31^, ^34^, ^36^, ^37^, ^38^, ^40^, ^43^, ^45^, ^46^, ^48^, ^53^, ^55^ Abnormal nerve conductive velocity accounts for 64.71% (11/17) (Fig. 4).

*PLP1* gene is located in Xq22.2 containing 7 exons and 6 introns, and it spans approximately 17 kb.^12^ It encodes proteolipid proteins PLP1 and one spliced isoform DM20, which account for more than 50% of the total protein mass of myelin in central nervous system (CNS).^56^ The pathogenesis mechanism lies in misfolded protein accumulation in endoplasmic reticulum (ER), toxic overexpression, and loss function of PLP1.^57^, ^58^, ^59^ DM20 contains 242 amino acids, which differs from PLP1 (277 aa) in a deletion of 35 amino acids (117–151) from the major hydrophilic domain.^60^ PLP1 and DM20 play an important role in stabilizing and maintaining the myelin sheath and in the development of oligodendrocytes precursors.^56^, ^61^ Genomic deletions of PLP1 directly lead to the physically fragile myelin sheath, which is susceptible to subsequent demyelination.^62^ While, overexpression of PLP1 also results in perturbed myelination and reduced viability of oligodendrocytes via cholesterol and PLP1 accumulation as well as mis‐trafficking of raft components.^63^ Some female heterozygous carriers may develop late‐onset gait disturbance, which is probably due to skewed inactivation of the wild‐type allele on the X chromosome.^10^, ^52^ Indeed, female carriers with a gross deletion in *PLP1* are likely to present with EONDT (severe developmental delay, intellectual disability, and behavioral abnormalities).^18^, ^19^ Furthermore, the phenotypic heterogeneity of PLP1‐related disorders might be related with variable genetic background, the contribution of genetic modifiers of PLP1 as well as the environmental factors.^64^, ^65^, ^66^

Excitingly, potential therapeutic targets for PLP1‐related disorders are emerging. Morpholino antisense oligomers could significantly shift alternative splicing toward *PLP1* expression in oligodendrocyte cell line.^67^ Colony‐stimulating factor 1 receptor (CSF‐1R) inhibitor PLX3397 could significantly reduce resident microglia and T‐lymphocyte recruitment in the CNS of two *PLP1* mutant mouse models.^68^, ^69^ In addition, umbilical cord blood transplantation could delay the PMD's progression and improve myelination.^70^ Furthermore, cytotoxic drugs VX680 or 5azadC successfully reversed the abnormal X‐chromosome inactivation and restored expression of the wild‐type allele in the female carrier‐derived lymphoblastoid cell line.^52^

## Conclusion

Overall, our study reported three families with SPG2, in combination with cognitive impairment, WML, with or without ataxia and tremor. The kinetic tremor in upper limbs was observed in 2/3 families, suggesting the spectrum of PLP1‐related disorders is still expanding.

## Data Analysis

Li Yao, Wotu Tian, and Li Cao, Department of Neurology, Shanghai Sixth People's Hospital Affiliated to Shanghai Jiao Tong University School of Medicine, Shanghai, 200233, China.

## Author Contributions

Li Yao and Zeyu Zhu contributed to data collection, analysis, and drafted the manuscript. Chao Zhang contributed to data collection. Wotu Tian and Li Cao contributed to study design and conceptualization, data acquisition, data analysis, interpretation of data, and manuscript revision.

## Conflict of Interest

The authors declare that they have no conflict of interest.

## Disclosure

Prof. L Cao is in charge of National Natural Science Foundation of China (No. 81870889 and 82071258). Dr. Tian is in charge of National Natural Science Foundation for Young Scholars of China (No. 82201398), China Postdoctoral Science Foundation (No. 2022 M712117), Shanghai Pujiang Program (22PJD052), and Young Scholar Cultivation Project of Basic Scientific Research in Shanghai Sixth People's Hospital Affiliated to Shanghai Jiao Tong University School of Medicine (No. YNQN202224). The other co‐authors report no disclosures relevant to the manuscript.

## Data Availability

The original dataset used and analyzed for this study is available from the corresponding author on reasonable request.
